# *upSET*, the *Drosophila* homologue of SET3, Is Required for Viability and the Proper Balance of Active and Repressive Chromatin Marks

**DOI:** 10.1534/g3.116.037788

**Published:** 2017-01-04

**Authors:** Kyle A. McElroy, Youngsook L. Jung, Barry M. Zee, Charlotte I. Wang, Peter J. Park, Mitzi I. Kuroda

**Affiliations:** * Division of Genetics, Brigham and Women’s Hospital, Boston, Massachusetts 02115; † Department of Genetics, Harvard Medical School, Boston, Massachusetts 02115; ‡ Department of Molecular and Cellular Biology, Harvard University, Cambridge, Massachusetts 02138; § Center for Biomedical Informatics, Harvard Medical School, Boston, Massachusetts 02115

**Keywords:** *Drosophila*, chromatin, heterochromatin, position effect variegation, *upSET*, SET3, MLL5

## Abstract

Chromatin plays a critical role in faithful implementation of gene expression programs. Different post-translational modifications (PTMs) of histone proteins reflect the underlying state of gene activity, and many chromatin proteins write, erase, bind, or are repelled by, these histone marks. One such protein is UpSET, the *Drosophila* homolog of yeast Set3 and mammalian KMT2E (MLL5). Here, we show that UpSET is necessary for the proper balance between active and repressed states. Using CRISPR/Cas-9 editing, we generated S2 cells that are mutant for *upSET*. We found that loss of UpSET is tolerated in S2 cells, but that heterochromatin is misregulated, as evidenced by a strong decrease in H3K9me2 levels assessed by bulk histone PTM quantification. To test whether this finding was consistent in the whole organism, we deleted the *upSET* coding sequence using CRISPR/Cas-9, which we found to be lethal in both sexes in flies. We were able to rescue this lethality using a tagged *upSET* transgene, and found that UpSET protein localizes to transcriptional start sites (TSS) of active genes throughout the genome. Misregulated heterochromatin is apparent by suppressed position effect variegation of the *w^m4^* allele in heterozygous *upSET*-deleted flies. Using nascent-RNA sequencing in the *upSET*-mutant S2 lines, we show that this result applies to heterochromatin genes generally. Our findings support a critical role for UpSET in maintaining heterochromatin, perhaps by delimiting the active chromatin environment.

Chromatin, the environment in which DNA is packaged, and transcription, the molecular dance to faithfully express the genic content of DNA, are intimately linked. As such, chromatin proteins exert a strong influence on the timing, patterning, and level, of gene expression. This influence can be in the form of direct binding to histones/nucleosomes or DNA, the post-translational modification (PTM) of histones, or DNA modification. One family of chromatin-associated proteins that post-translationally modify histones is the SET domain-containing proteins. The SET domain catalyzes methylation of histone tails. Different SET domain-containing proteins create unique histone modifications, which are associated with different forms of active and repressed chromatin environments.

Of the family of SET domain proteins, one paralog is a notable exception to this paradigm. The *Drosophila* protein UpSET and its homologs Set3 in yeast and KMT2E (henceforth referred to as MLL5) in mammals are not known to have histone-modifying activity (Pijnappel *et al.* 2001; Madan *et al.* 2009; Rincon-Arano *et al.* 2012). In fact, conserved catalytically important residues in the SET domain are mutated in UpSET/Set3/MLL5, suggesting that protein function must not rely on the competence of the SET domain (Madan *et al.* 2009). Rather than catalyzing histone methylation, these proteins have been characterized in yeast and flies to form complexes with histone deacetylases (HDACs) (Pijnappel *et al.* 2001; Rincon-Arano *et al.* 2012).

Set3 is a nonessential gene in yeast (Pijnappel *et al.* 2001), with a mutant phenotype of defective transcription kinetics when cells are metabolically challenged (Wang *et al.* 2002; Kim *et al.* 2012). Set3 complex (Set3C) was also recently tied to the DNA damage response operating under a model of altered histone acetylation dynamics (Torres-Machorro *et al.* 2015). MLL5 in mammals has been tied to several different cellular processes, including hematopoesis (Heuser *et al.* 2009; Madan *et al.* 2009; Zhang *et al.* 2009), cell cycle progression (Deng *et al.* 2004; Cheng *et al.* 2008), oncogenesis (Emerling *et al.* 2002), and DNA methylation (Yun *et al.* 2014), though its exact mechanistic role or binding partners in these diverse functions have not been fully resolved. In *Drosophila*, *upSET^e00365/e00365^* flies were described to be viable, but with a female fertility defect due to derepression of transposable elements in the ovary (Rincon-Arano *et al.* 2012).

We previously identified the UpSET protein (CG9007) as a top interactor with the MSL3 protein by a cross-linked affinity purification technique (Wang *et al.* 2013). MSL3 is a chromodomain protein that is a core constituent of the Male-specific lethal (MSL) dosage compensation complex in *Drosophila*. The five genetically defined MSL proteins (Male-specific Lethal-1, -2, -3, Maleless, and Males-absent-on-first), and two redundant noncoding RNAs (*RNA on X -1 and -2*) localize specifically to the male X to create a unique chromatin environment, and boost expression of active genes (for review, see Lucchesi and Kuroda 2015). The chromatin environment that is created by the MSL complex is catalyzed by the Males-absent-on-first (MOF) protein, which acetylates histone 4 on lysine 16 (H4K16ac). This modification has a very stereotypical pattern near TSSs on autosomes, and across the female genome. In contrast, on the male X, H4K16ac is enriched on gene bodies, reflecting the localization of the MSL complex, and its putative function in transcriptional elongation.

The potential interaction between UpSET, a member of an HDAC complex, and MSL3, a member of the MSL complex that makes an acetyl mark, led us to create *upSET* mutant S2 cell lines using the CRISPR/Cas-9 system. We surveyed bulk histone PTM levels in these cells to test for a global effect on the H4K16ac dosage compensation-associated mark, but instead observed that the relative amounts of the heterochromatin mark histone H3 lysine 9 dimethyl (H3K9me2) were strongly reduced. To investigate this potential heterochromatin association, we created an *upSET* deleted *Drosophila* fly line using the CRISPR/Cas-9 system for genome engineering coupled with a homologous recombination donor. We observed *upSET^DsRed+{^*^Δ^*^upSET}/DsRed+{^*^Δ^*^upSET}^* to be lethal in both males and females, confirming a role independent of, or broader than, regulation of male-specific dosage compensation, and in contrast to a previous report of homozygous mutant viability (Rincon-Arano *et al.* 2012). *upSET*^Δ^*^DsRed{^*^Δ^*^upSET}/+^* heterozygotes exhibit a suppressor of variegation phenotype, suggesting that heterochromatic silencing is influenced by UpSET dosage. Furthermore, we also detected perturbation of heterochromatin gene expression in *upSET* mutant cell lines using nascent-RNA sequencing. Together with previous studies, our results suggest that heterochromatin and heterochromatin-embedded genes are particularly sensitive to a balance of chromatin-modifying activities, regulated in part by the UpSET protein.

## Materials and Methods

### Generating upSET mutant S2 cells and flies

S2 cells and mutant S2 lines were maintained at 26° in Schneider’s medium supplemented with 10% FBS and 1% antibiotic/antimycotic (Gibco). Mutant S2 cells were generated using the CRISPR/Cas-9 system essentially as described (Housden *et al.* 2015). Guide RNA sequences were obtained from the *Drosophila* RNAi Screening Center (DRSC)’s sgRNA design tool (www.flyrnai.org/crispr2). Oligonucleotides of the appropriate gRNA sequence were ordered from IDT (Table S1), with additional bases to allow for ligation into the *Bbs*I site of the pL018 plasmid (a gift from N. Perrimon), which also expresses Cas-9. S2 cells were transfected using Effectene (Qiagen) using 40 ng of an actin::RFP marker plasmid (a gift from T. Wu), and 360 ng total of the appropriate pL018 construct, either singly or in combination with other pL018 constructs. At 4 d after transfection, single cells in the top 10% of RFP^+^ cells (typically top ∼3% of total population) were sorted by FACS into conditioned medium in 96-well plates. Colony growth from single S2 cells was observed after 2–3 wk. Independent lines were expanded and tested for mutations by HRMA (high resolution melt assay) using Precision Melt Supermix (Bio-Rad). Lines scoring well in the HRMA had the gRNA target region amplified by PCR using flanking primers (Table S1), and the resulting product was subcloned into the pCR4Blunt-TOPO vector (Invitrogen). To identify the molecular lesion, five bacterial colonies per S2 line were sequenced by Genewiz. Lines with mutations that introduce frameshifts or deletions of the start codon and adjacent sequence were used for subsequent experiments.

*upSET*-directed gRNA sequences were as follows:

upset1: 5′-AACCGAGTCGTGACTGGACATGG-3′
upset3: 5′-AGGCGCGATGCCGTCTGATTAGG-3′
upset5: 5′-TGGCCAGGCGCAGTAGTAATAGG-3′
upset7: 5′-ACAGCAGATCAGCCTACCGCAGG-3′

Mutant flies were generated by injecting gRNA constructs, and a homologous recombination donor, into *w*; *w^−^{nos-cas9}/CyO* embryos [a gift from N. Perrimon, see also Housden *et al.* (2014) for general guidelines for *Drosophila* CRISPR/Cas-9]. Injections were made using glass capillary needles through the intact chorion essentially as described (Miller *et al.* 2002). The gRNA constructs were designed as above, and oligos were ligated into the pU6-BbsI-chiRNA plasmid (#45946; addgene) as described (Gratz *et al.* 2013). The homologous recombination donor was constructed using 1 kb of genomic sequence flanking the 5′ and 3′ gRNA cut sites inserted into the pDsRed-attP plasmid (#51019; addgene) (Gratz *et al.* 2014), which expresses the synthetic marker 3×P3-DsRed in the adult eye. Genome (R6.12) coordinates, 5′ homology arm: 3L:14006860..14007859; 3′ homology arm: 3L:14017877..14018876. Adults resulting from the injections were outcrossed to *yw* flies, and their progeny were screened for the fluorescent DsRed marker. DsRed-positive progeny were crossed to *yw*; *TM3/TM6* flies to generate balanced stocks of *yw*; +; *DsRed^+^{*Δ*upSET}/TM3* or *yw*; +; *DsRed^+^{*Δ*upSET}/TM6*.

In order to mitigate any interference of the DsRed marker with eye phenotype scoring in position effect variegation experiments, we excised the 3×P3-DsRed cassette using Cre recombination with the flanking loxP sites present in the pDsRed-attP vector. Cre recombinase was introduced from the *yw*; *MKRS*,*{hs-FLP}/TM6B*,*{Crew}*,*Tb* line (#1501; Bloomington). *yw*; *DsRed^+^{*Δ*upSET}/TM6B*,*{Crew}Tb* progeny were crossed to a third chromosome balancer line to establish balanced stocks of *yw*; Δ*DsRed{*Δ*upSET}/TM3* or *TM6*. Successful mobilization of the DsRed cassette was confirmed in all crosses by visual inspection.

### Cloning and transgenesis for the upSET-BioTAP allele

The UpSET-BioTAP allele was constructed using the pRedET recombineering system (GeneBridges K002). The genomic region of *upSET* was transferred to the pFly (aka pGS-mw) vector, and injected into flies for site-specific integration at 53B2 on the second chromosome (#9736; BestGene stock). The resulting flies, *yw*; *UpSET-BioTAP/UpSET-BioTAP*, were crossed into the *DsRed^+^{*Δ*upSET}* background. *DsRed^+^{*Δ*upSET}*-homozygous flies carrying one or two copies of the UpSET-BioTAP allele were used for one-step ChIP (see below).

Presence of WT *upSET* or the BioTAP-tagged-*upSET* transgene was assessed by PCR from genomic DNA isolated from two to three female flies of the specific genotype. A 544 bp product from wild type (WT), and a 1210 bp product from the BioTAP-tagged *upSET* construct are obtained when using the following primer pair:

KAM201: 5′-gctgcacatgtttgatgataagc-3′
KAM202: 5′-gtgcaagctcatactttatgcgc-3′

### Bulk histone purification and mass spectrometry

Bulk histones were salt-acid extracted from S2 cell lines using 2M NaCl and 0.4N H_2_SO_4_ following cell lysis with RIPA buffer. Histone proteins were precipitated using trichloroacetic acid (TCA), and resuspended in 25 mM sodium bicarbonate. Resuspended histone proteins were treated with propionic anhydride (Sigma Aldrich) for bottom-up peptide analysis by liquid chromatography–mass spectrometry as described in a previous report (Zee *et al.* 2016a,b).

### Small scale one-step ChIP-seq from BioTAP-tagged embryos

To assess the genomic localization of BioTAP-tagged proteins from a small scale of embryos, immunoprecipitation using only the proteinA moieties of the tag were performed. Using a protocol essentially described elsewhere (Alekseyenko *et al.* 2008), 0.1 g of embryos was collected, and disrupted using a motorized pestle. Formaldehyde was added to 1% final concentration, and incubated for 15 min at room temperature. Following quenching of the reaction with glycine and washing, fixed material was sonicated in RIPA buffer using a Bioruptor, four cycles of 30 sec on/30 sec off on the high setting. Sonicated material was supplemented with TritonX-100 to 1%, Sodium DOC to 0.1%, and NaCl to 140 mM, and debris was cleared by centrifugation. Chromatin was aliquoted and stored at −80° until IP. For ChIP, 20–30 μl of IgG agarose slurry per IP were washed in RIPA buffer, and incubated with chromatin overnight. Bound immunocomplexes were washed 5× with RIPA (140 mM NaCl; 10 mM Tris, pH 8; 1 mM EDTA, pH 8; 1% Triton; 0.1% SDS; and 0.1% sodium deoxycholate), once with LiCl buffer (250 mM LiCl; 10 mM Tris pH 8; 1 mM EDTA, pH 8; 0.5% NP40; and 0.5% sodium deoxycholate), twice with TE, and finally resuspended in TE. Input and IPs were treated for 30 min with RNase at 37°, then overnight with the addition of proteinase K and SDS (0.5% final), and crosslinks were reversed for 6 hr at 65°. IP samples were supplemented with NaCl to 140 mM final, and both IP and input samples were extracted with an equal volume of 25:24:1 phenol:chloroform:isoamyl alcohol. To maximize recovery in IPs, the organic fraction was extracted with TEN140 (TE + 140 mM NaCl), and pooled with the initial aqueous phase. All samples were then extracted with an equal volume of 24:1 chloroform:isoamyl alcohol, and precipitated overnight at −80° with sodium acetate and ethanol, in the presence of glycogen. The entirety of the precipitated IP-DNA and ∼200 ng of input DNA were used to create high-throughput sequencing Illumina libraries using the NEBNext ChIP-seq kit (NEB 6240). Prior to library amplification, size selection was achieved using a 2% agarose gel (50111; Lonza). Sequencing was performed at the Tufts Genomics Core.

### ChIP-seq analysis

The adaptor sequences were trimmed with Cutadapt ver. 1.2.1 (Martin 2011). The reads were aligned to the *Drosophila* genome (dm3 assembly) using Bowtie ver. 12.0 (Langmead *et al.* 2009) with a unique mapping option (-m 1). Only uniquely aligned reads were used for analysis. The input normalized fold enrichment profiles were generated using the *get.smoothed.enrichment.mle* function of the SPP R package (Kharchenko *et al.* 2008), with a step size of 20 bp, and Gaussian kernel bandwidth of 150 bp. The profiles were normalized by the background scaling method. For metagene plots, the regions in the gene body except for 500 bp margins of 5′-end and 3′-end were scaled and averaged after merging two replicates. Only genes >1.5 kb in length, and >1 kb away from adjacent genes, were included in the metagene analysis. To estimate gene expression, RNA-seq samples profiled by modENCODE consortium were used for S2 cell and 14–16 hr embryos (Gerstein *et al.* 2014). FPKM = 1 was used as a threshold for expressed genes. To detect significantly enriched peaks, the *get.broad.enrichment.clusters* function of the SPP R package was used, with a window size of 1 kb and *z*-score threshold of 3. For downstream analysis based on peaks, we used the significant peaks from the second UpSET-BioTAP replicate, which has a better signal-to-noise ratio. The genomic annotation for UpSET was performed using CEAS (Shin *et al.* 2009). For chromatin annotation, the chromatin segmentations were obtained from previous studies of S2 (Kharchenko *et al.* 2011) and embryos (Ho *et al.* 2014).

### Position effect variegation of the w^m4^ allele

Virgin females of the genotype *w^m4^/w^m4^*; +; + were crossed to males of the genotype *yw*; Δ*DsRed{*Δ*upSET}/TM3*,*Sb or yw*; +; +. Resulting progeny of this cross were sorted to the appropriate third chromosome genotype by balancer chromosome markers. These flies were maintained at 24° for 3 d, after which variegation of eye pigmentation was assessed. The extent of eye pigmentation was scored into three classes.

### Nascent-RNA-seq from S2 cells

Nascent-RNA sequencing was done using a urea-based method (Ferrari *et al*. 2013) essentially as described (Alekseyenko *et al.* 2015). In short, 1 × 10^7^ S2 cells were collected by centrifugation at 300 × *g* at 4°, and homogenized in CKS buffer + SUPERase•In RNase inhibitor (Ambion AM2696) + ProteaseArrest (786-108; G-Biosciences) with three strokes through a 25 G needle. Nuclei were collected by centrifugation, and resuspended in CF buffer + RNasin. NUN buffer was added and samples were vortexed ∼30 sec until a wispy, filamentous precipitate was apparent. This precipitate was spun down and washed three times with NUN buffer. Samples were then treated with proteinaseK in CF buffer + 0.5% SDS at 55° for 30 min. Samples were then passed through a 25 G needle five times to disrupt the chromatin, and incubated an additional 30 min at 55°. Samples were extracted twice with 25:24:1 phenol:chloroform:isoamyl alcohol, once with 24:1 chloroform:isoamyl alcohol, and ethanol precipitated overnight in the presence of glycogen (AM9510; Ambion). Resulting nucleic acids were treated with RNase-free TURBO DNase (AM2238; Ambion) for 30 min at 37°, with proteinase K + SDS for an additional 5 min at 37°, and then extracted once each with 25:24:1 phenol:chloroform:isoamyl alcohol, and 24:1 chloroform:isoamyl alcohol. Nascent-RNA was ethanol precipitated overnight except without the addition of glycogen. Illumina sequencing libraries were constructed using the NEBNext Ultra Directional RNA Library kit (NEB 7420).

### Nascent-RNA-seq analysis

The reads were mapped as described above. The tag density profiles were generated using *get.smoothed.tag.density* function of SPP R package, with a Gaussian kernel of 100 bp, and a step size of 10 bp after library size normalization. To compare the enrichment values between the mutant and control, the reads were separated to sense and antisense transcripts. The fold change, and significantly changed regions in transcription between conditions, were determined using EdgeR (Robinson *et al.* 2010) after TMM (trimmed mean normalization method) (Ross-Innes *et al.* 2012). Heterochromatic and euchromatic genes were defined using the heterochromatin, and euchromatin boundary information for each chromosome obtained from a previous study based on H3K9me2 enrichment levels (Riddle *et al.* 2011). To access the significance for the portions of upregulated genes between groups, a bootstrap method was used (*n* = 1000).

### Data availability

Cell and fly lines are available upon request. Mass Spectrometry raw data files are available through Harvard Dataverse (https://dataverse.harvard.edu/) at doi: 10.7910/DVN/LFGYUV. Gene expression (nascent-RNA) and ChIP-seq data are available at GEO accession number GSE90703.

## Results

### CRISPR-engineered S2 cell lines tolerate inactivating upSET mutations

**Figure 1:**
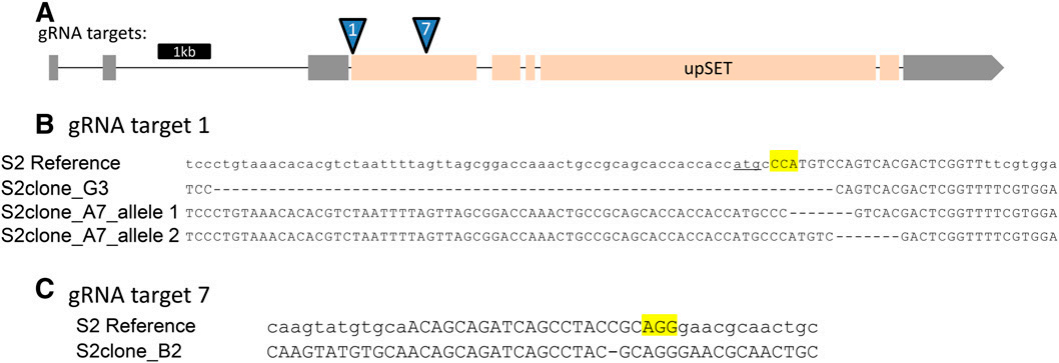
Generation of *upSET* mutant S2 lines. (A) Schematic for the *upSET* gene locus. Noncoding exons are in gray, while coding exons are in light orange. Locations of guide RNA targets for Cas9 are indicated. (B) Molecular lesions generated around the *upSET* gRNA #1 target site, located just downstream of the *upSET* start codon (underlined). In the S2 reference sequence, the gRNA target is in capital letters, and the PAM is highlighted. Clone G3 has a homozygous 67 bp deletion, removing the start codon, and 10 additional coding base pairs, as well as 54 bp of the adjacent sequence. Clone A7 has two separate 7 bp deletions, both resulting in frameshift mutations, and predicted truncations in the protein product. (C) As in (B), in the S2 reference sequence, the gRNA target is in capital letters and the PAM is highlighted. The molecular lesion generated around the *upSET* gRNA #7 target site in clone B2 carries a 1 bp deletion, resulting in a frameshift after 367 amino acids of the wild-type UpSET protein sequence, and a predicted 436 amino acid product.

In order to assess the molecular effects of the loss of UpSET, specifically in male cells, we generated S2 cell lines stably carrying *upSET* mutations. S2 cells are a male *Drosophila* cell line which is highly polyploid. We reasoned that these cells may tolerate perturbed chromatin states better than the whole organism, given their tolerance of their nondiploid genome. The ploidy of the genome introduces its own challenges for genome engineering, yet we saw that mutations typically went to fixation when we used the CRISPR/Cas-9 system (Housden *et al.* 2015). We cotransfected S2 cells with an RFP-expressing marker plasmid, and a plasmid coexpressing both the Cas-9 protein and one or more of several different guide RNAs directed toward the *upSET* gene (Figure 1A). To isolate single clones, RFP-positive transfected cells were sorted into 96-well plates by FACS. Following regrowth from these single cells, we identified lines with putative mutations by high-resolution melt assays (HRMA) (Bassett *et al.* 2013). Statistically significant hits were further analyzed by Sanger sequencing to identify the nature of the molecular lesions at the gRNA target site. In this way, we were able to isolate three *upSET* clonal mutant lines that lack a wild-type UpSET open reading frame (Figure 1, B and C).

### Bulk histone PTM analysis in upSET mutant S2 cells reveals a perturbed chromatin state

To explore the potential role of UpSET in chromatin and gene expression, we sought to obtain a comprehensive assessment of all histone post-translational modifications that were altered by the loss of *upSET*. We isolated bulk histones from S2 cells, and all three *upSET* mutant cell lines, using a salt/acid extraction method (Zee *et al.* 2016a). To quantitatively recover histone peptides with their PTM state intact for mass spectrometry analysis, histones were derivatized in solution with a protecting group that allows recovery of histone peptides from reverse phase chromatography. Using the mass difference and relative elution order between the protecting group and various modifications (acetylation, methylation, etc.), we were able to quantify the relative abundance of a given histone PTM with respect to all observable modified forms within the same tryptic peptide backbone within each sample. As a positive control for our methodology, we also assessed the histone modifications in S2 cells treated with the general HDAC inhibitor, sodium butyrate (S2but), which should result in global accumulation of acetylation (Candido *et al.* 1978).

**Figure 2:**
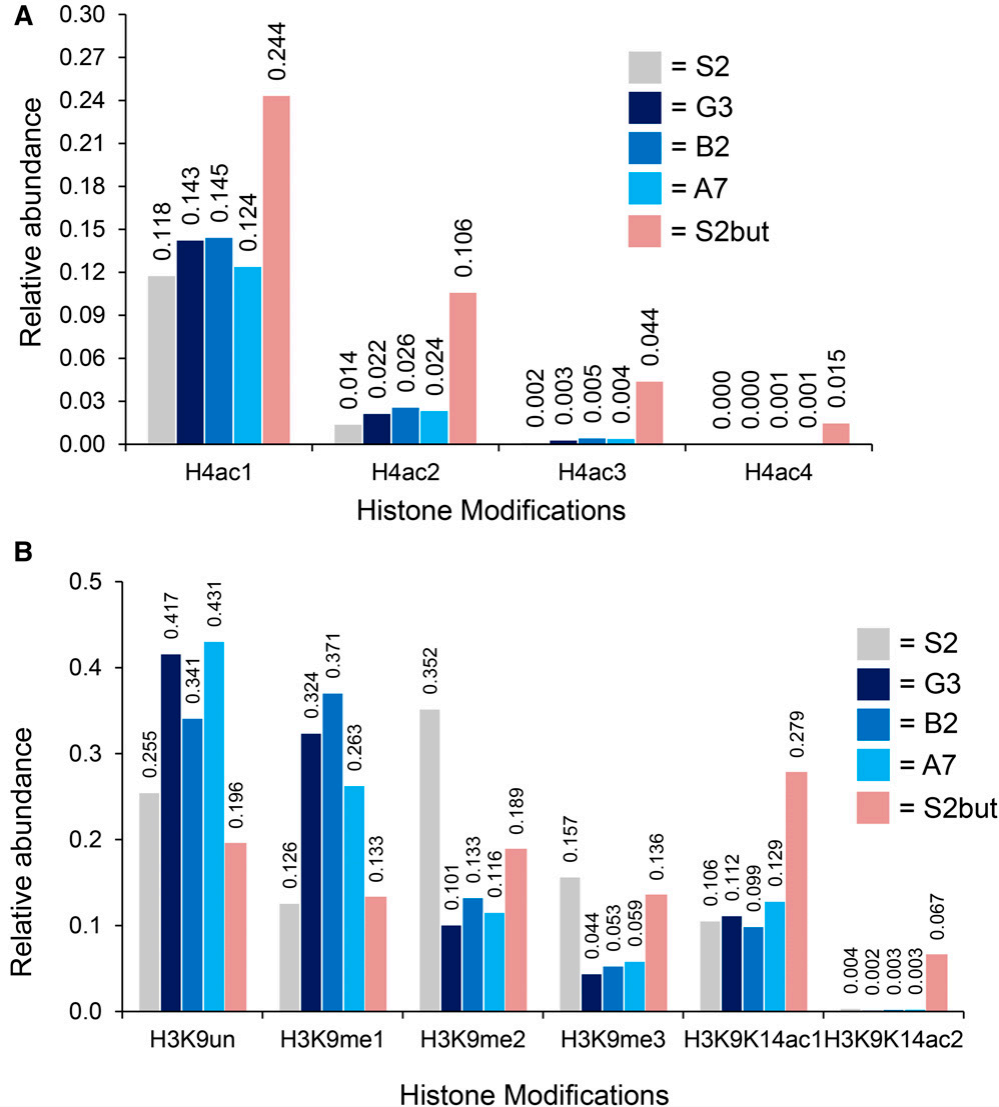
Analysis of histone post-translational modifications from bulk histones in *upSET* mutant cells. (A) Relative quantification (1.0 = 100%) of H4K5K8K12K16 mono-, di-, tri-, and tetra-acetyl patterns in *upSET* mutant cell lines (G3, B2, and A7), butyrate treated cells (S2but), and the parental S2 line. The patterns of the mutant or butyrate treated cells differ from the parental cell line. Sodium butyrate inhibits deacetylases, resulting in accumulation of acetylation marks. (B) Relative quantification (1.0 = 100%) of H3K9K14 PTM patterns in *upSET* mutant cell lines (G3, B2, and A7), butyrate treated cells (S2but), and the parental S2 line. The H3K9me2/me3 marks are depleted from *upSET* mutant S2 cells, suggestive of an effect on heterochromatin.

We observed a slight increase in H4-monoacetylation on the peptide that contains lysines at residues 5, 8, 12, and 16 in *upSET* mutant cells, and a larger increase in the butyrate-treated cells (Figure 2A). We obtained similar results in replicates (Supplemental Material, Figure S1A and File S1). We also found an increase in H3K4me1, as compared to unmodified H3K4 (Figure S2). However, a comprehensive view of the modification state at this residue was not possible, due to the hydrophillic nature of the H3K4me2/me3 peptides, which precludes their recovery during desalting prior to liquid chromatography-mass spectrometry.

Unexpectedly, the largest relative changes in modifications on total histones in the *upSET* mutant cells were in H3K9me2 and H3K9me3 levels, which were greatly diminished compared to wild-type S2 cells, with a concomitant increase in monomethylated and unmethylated H3K9 (Figure 2B). We obtained similar results in replicates (Figure S1B). In contrast, cells treated with S2but also experienced a drop in H3K9me2/3 levels, however with a concomitant increase in H3K9K14 monomethylation or diacetylation. H3K9me2 is a modification known to be enriched in heterochromatin (Ebert *et al.* 2004). The HP1a protein, which is critical for heterochromatin formation, interfaces with this mark via its chromodomain (Platero *et al.* 1995; Jacobs and Khorasanizadeh 2002). These data suggest an as-yet undetermined role for UpSET in heterochromatin.

### upSET is an essential gene in Drosophila

**Figure 3:**
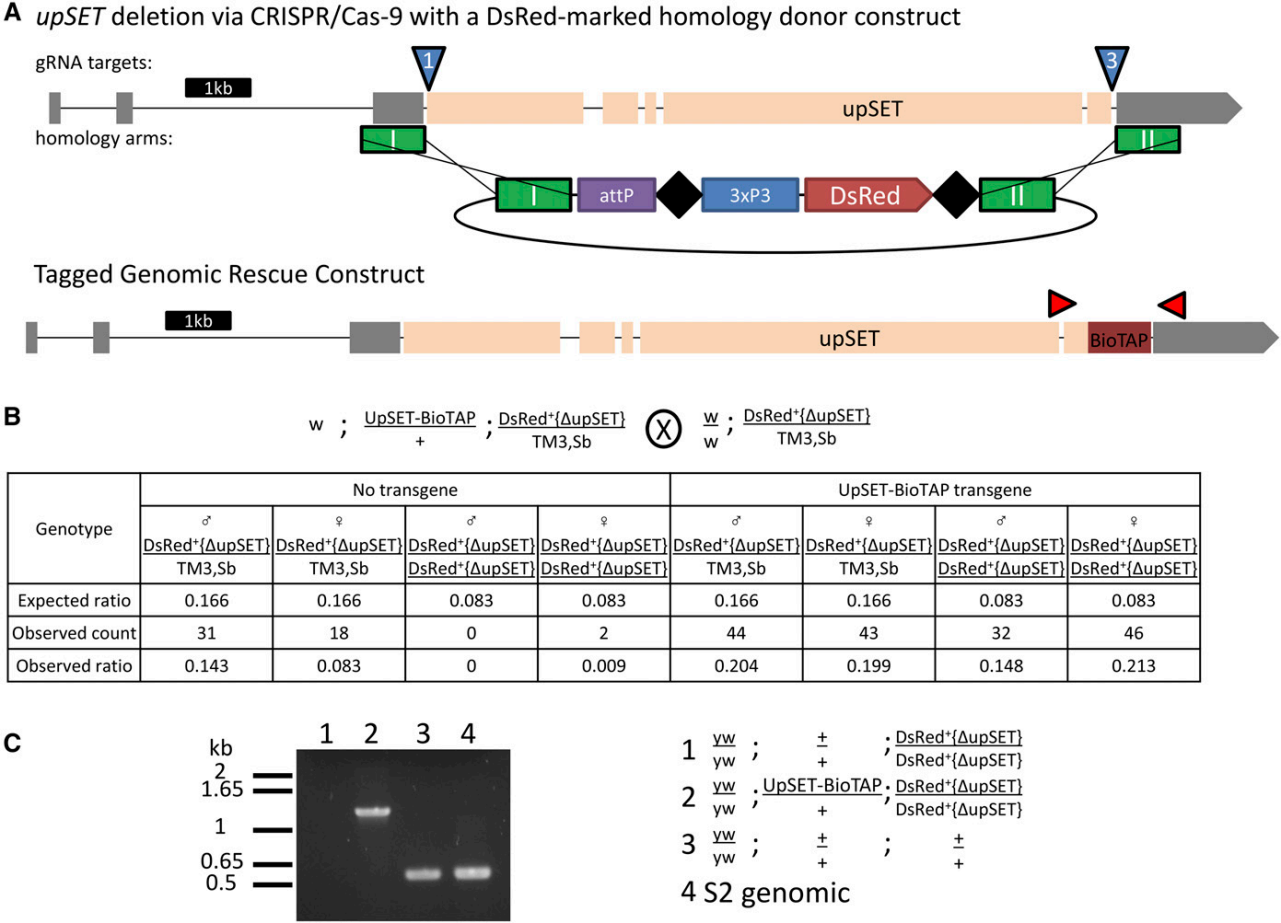
*upSET* is an essential gene in *Drosophila*. (A) The *upSET* gene, as depicted in Figure 1, with the location of guide RNA targets (blue triangles), homologous recombination donor arms (green rectangles), and the structure of the DsRed donor construct indicated. The BioTAP-tag insertion in a separate transgenic rescue construct shown below is indicated in crimson, with flanking PCR primers (red, not to scale). (B) *upSET* is homozygous lethal, but is rescued by the UpSET-BioTAP transgene (compare counts in columns three and four to columns seven and eight). Cross scheme is shown above the table. Table contains the expected ratio of adult progeny per genotype, as well as the observed count and ratio of these genotypes. (C) PCR analysis reveals that homozygous escapers lack the 544 bp wild-type product. The rescued flies produce only the band corresponding to the tagged construct.

The previous characterization of *upSET* mutant flies utilized the only two lines then available, which carry a *P*-element and Minos insertion in the *upSET* gene, respectively (Rincon-Arano *et al.* 2012). However, the insertions carried by both of these lines leave the coding sequence largely intact. While Western blotting suggested no residual protein, given the possibility that those alleles could in fact be hypomorphic instead of complete loss-of-function, we sought to create an *upSET* deletion allele with the coding sequence removed from the genome. To accomplish this, we turned to the versatile CRISPR/Cas-9 genome engineering system. We coinjected *w*; *w^−^{nos-cas9}/CyO* embryos, which express Cas-9 in the germ line, with two guide RNA constructs, and a homologous recombination donor marked with 3xP3-DsRed (Figure 3A). We isolated balanced flies that carried the DsRed marker, and confirmed the loss of the entire predicted *upSET* coding sequence by PCR. We found that homozygosity for the *upSET* deletion is lethal, with a low escape rate. The few escapers that were recovered were sickly, and did not reproduce with *yw* mates. We were able to rescue this lethality with an UpSET-BioTAP transgene (Figure 3B). PCR from rescued individuals confirmed that no wild-type *upSET* DNA remained (Figure 3C). Taken together, these results suggest that, contrary to previous reports, *upSET* is an essential gene in *Drosophila*, and that the previous alleles are hypomorphs, rather than *bona fide* complete loss-of-function.

### UpSET-BioTAP localizes to TSS of active genes by ChIP-seq

Our previous attempts to determine the localization of UpSET using the BioTAP-tagged transgene were unsuccessful in cell culture due to poor stability of the tagged protein in chromatin preparations (data not shown). We reasoned that more of the tagged protein would be incorporated into chromatin in the *upSET*-deleted background, and so we prepared chromatin for ChIP from 0.1 g of mixed 12–24 hr embryos carrying the UpSET-BioTAP transgene in the homozygous *DsRed^+^{*Δ*upSET}* background. We immunoprecipated UpSET-BioTAP using the protein-A moiety, and sequenced the resulting material.

**Figure 4:**
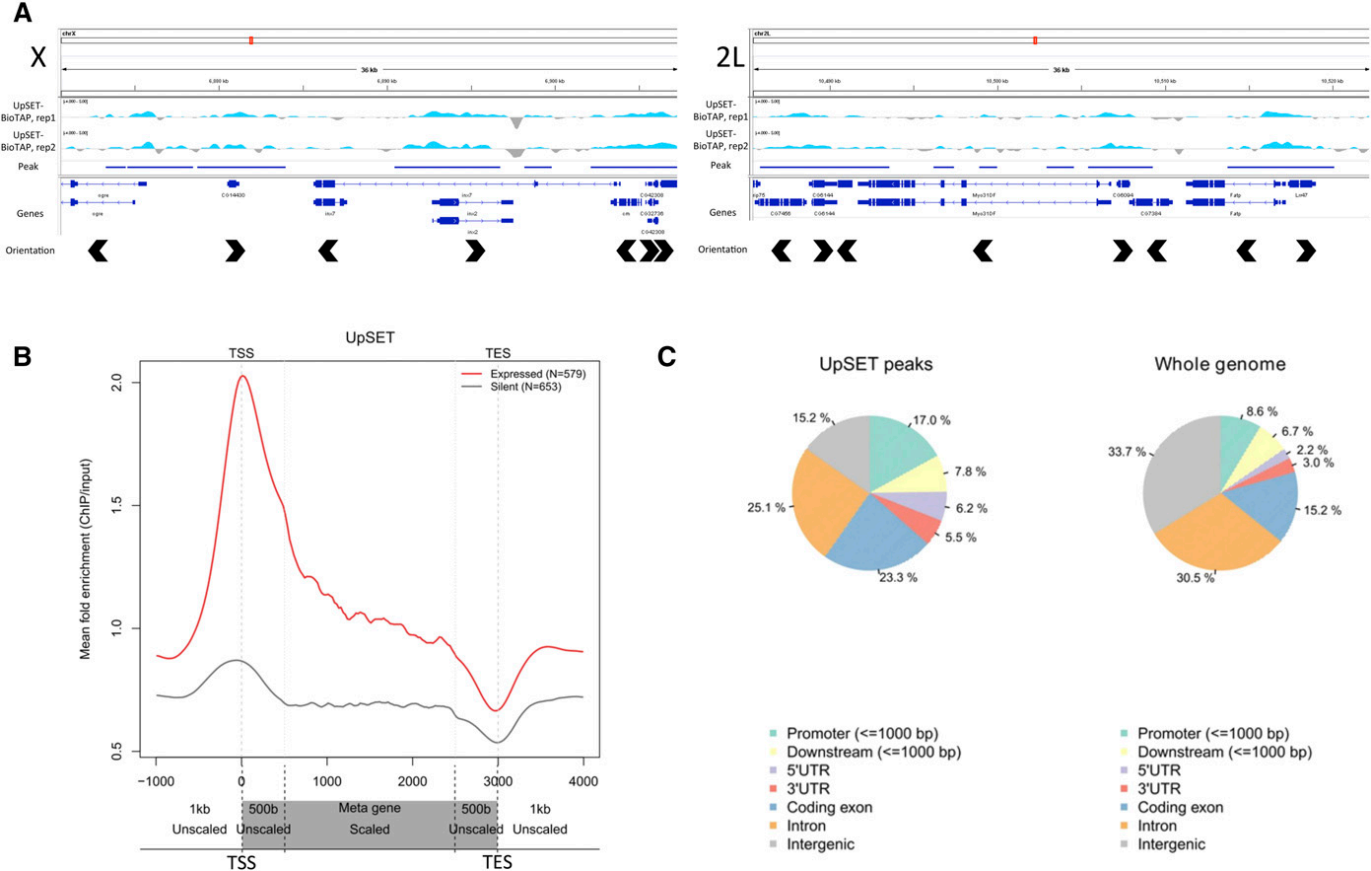
UpSET-BioTAP localizes to TSSs of active genes in embryos. (A) Two representative genome browser views of 36 kb regions of the X and 2L chromosomes displaying UpSET-BioTAP ChIP-seq tracks, the corresponding peak call used for downstream analysis, gene annotations, and gene orientations. There are no apparent differences between UpSET-BioTAP on the X chromosome and binding on the autosomes. The *y*-axes for these ChIP-seq tracks are on a log2-scale. (B) Metagene profile for UpSET-BioTAP binding. TSS, transcription start site; TES, 3′ terminal site. Gene bodies from TSS+500 bp to TES−500 bp are scaled. The 1 kb upstream and downstream, and 500 bp into the gene on either end are unscaled. UpSET-BioTAP shows a strong preference for binding at the TSS of active genes. The *y*-axis for this metagene is a linear scale. (C) UpSET-BioTAP peaks, as highlighted in (A), are enriched for genome regions annotated to be promoters, 5′UTR, and coding exons. Intron and intergenic regions are depleted compared to the whole genome background.

In agreement with previous localization data generated by UpSET Dam-ID in Kc cells (Rincon-Arano *et al.* 2012), we observed UpSET-BioTAP to localize to active genes by ChIP-seq (Figure 4, A and B). More specifically, in the BioTAP data, there is enrichment for UpSET-BioTAP ChIP peaks in regions carrying the chromatin signatures of TSS proximal and active elongation states (Ho *et al.* 2014) (Figure S3). This is further supported by comparing the overlap of UpSET-BioTAP ChIP peaks with different genome feature annotations. UpSET-BioTAP peak regions display a >2-fold enrichment over the genomic background for promoter and 5′ UTR regions, and are also enriched for coding exons (Figure 4C). Conversely, intron and intergenic regions are depleted from UpSET-BioTAP peaks when compared to the whole genome. When comparing the UpSET-BioTAP dataset to those publically available in the modENCODE project, we observe the highest correlation with Pol II-datasets (Figure S4), consistent with enrichment at the TSS of genes in the active chromatin context.

We sought to examine whether we could detect an X-specific localization pattern for UpSET-BioTAP as compared to the autosomal pattern, although, in mixed-sex embryos, any X-specific localization signal in males would be dampened. We did not detect any difference between UpSET-BioTAP localization at the TSS, or throughout the gene body, between X-linked and autosomal genes (Figure S5).

### Heterozygous loss of upSET influences position effect variegation

**Figure 5:**
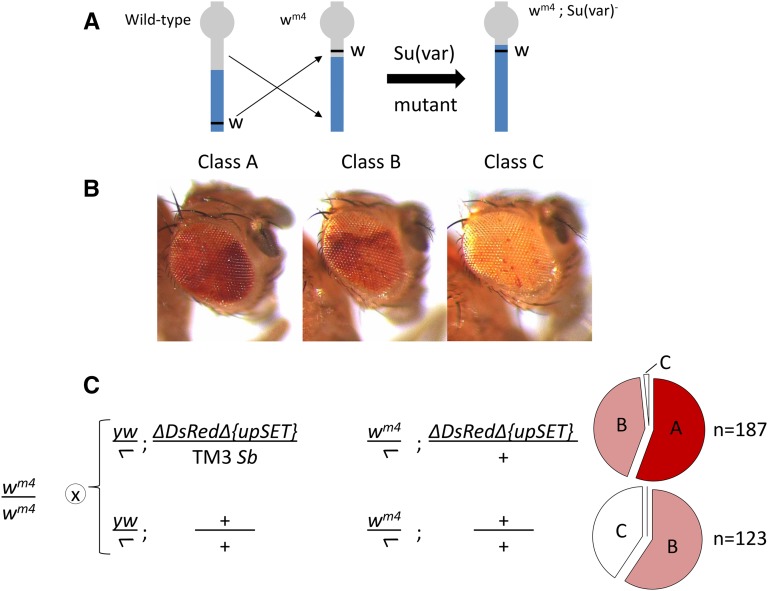
Heterozygous *upSET* mutants display a Suppressor of variegation [Su(var)] phenotype. (A) Schematic for the *w^m4^* allele (heterochromatin indicated in gray, euchromatin in blue, *white* locus in black). The *w^m4^* line has a *white* variegating phenotype due to juxtaposition of the *white* locus adjacent to heterochromatin on the X chromosome. Loss of repression of *white*, resulting in eyes with increased numbers of red pigmented cells, is thought to reflect destabilization of heterochromatin. (B) Representative images of the three classes of eye pigmentation into which flies were sorted. Class A has the highest proportion of red pigmentation, Class B has intermediate levels, and Class C is largely unpigmented. (C) Cross scheme for testing of the effect of heterozygous loss of *upSET* on position effect variegation. Male flies were scored at 3 d posteclosion into the three classes described above. In comparison to control males from a parallel cross, heterozygous loss of *upSET* leads to an increase in red pigmentation, skewing the population toward suppressed variegation (55.6% Class A, 42.8% B, 1.6% C for heterozygous Δ*DsRed{*Δ*upSET}* *vs.* 0% Class A, 59.3% B, 40.7% C for control males). Total number of progeny scored is indicated to the right of the pie chart.

To test whether the critical role for UpSET protein may be related to the maintenance of heterochromatin, we tested whether heterozygous loss of *upSET* would influence position effect variegation (PEV) of the *w^m4^* allele seen in the eyes of adult flies. The *w^m4^* allele carries an inversion on the X chromosome placing the *white* locus adjacent to heterochromatin, resulting in the apparent spreading of silencing into the locus and a variegating white-eyed phenotype (Figure 5A) (Elgin and Reuter 2013). Defects in heterochromatin components lead to expression of *white^+^* in a larger fraction of cells, thus larger sectors of red eye pigmentation. Loss-of-function mutants for the core components of heterochromatin score strongly in these assays and are collectively called suppressors of variegation [for example, Su(var)3–9, the primary enzyme responsible for H3K9 dimethylation in pericentric heterochromatin]. We scored the eye sectoring phenotypes of hemizygous *w^m4^* males heterozygous for Δ*DsRed{*Δ*upSET}* compared to hemizygous *w^m4^* males wild-type for the third chromosome obtained from a parallel cross. We observed that heterozygous loss of *upSET* results in suppression of variegation, that is, a larger number of flies with a greater extent of red pigmentation (Figure 5, B and C). This effect was also evident in female progeny, though there is a higher incidence of suppressed variegation in the control cross (Figure S6). Similarly, we observed suppression of PEV of a HS-lacZ reporter inserted on the Y chromosome (Lu *et al.* 1996) in heterozygous *DsRed^+^{*Δ*upSET}* larval salivary glands (data not shown). Suppression of PEV of a different *w^+^* reporter has also been reported (Rincon-Arano *et al.* 2013). These *in vivo* findings, along with our bulk analysis of histone PTMs in S2 cells, support the conclusion that UpSET plays a role in heterochromatin maintenance.

### Changes in transcription correlate with altered chromatin state

In order to assess whether global transcription might be affected by the altered chromatin states in *upSET* mutant cell lines, we utilized a urea-based method to sequence the nascently transcribed RNA associated with Pol II. We elected to isolate nascent RNA in order to limit our observations to changes in transcription, rather than in the steady state cytosolic pool of mRNA. Indeed, we observed that aberrant transcription occurred in all three *upSET* mutant S2 cell lines in comparison to the parental S2 cell line.

**Figure 6:**
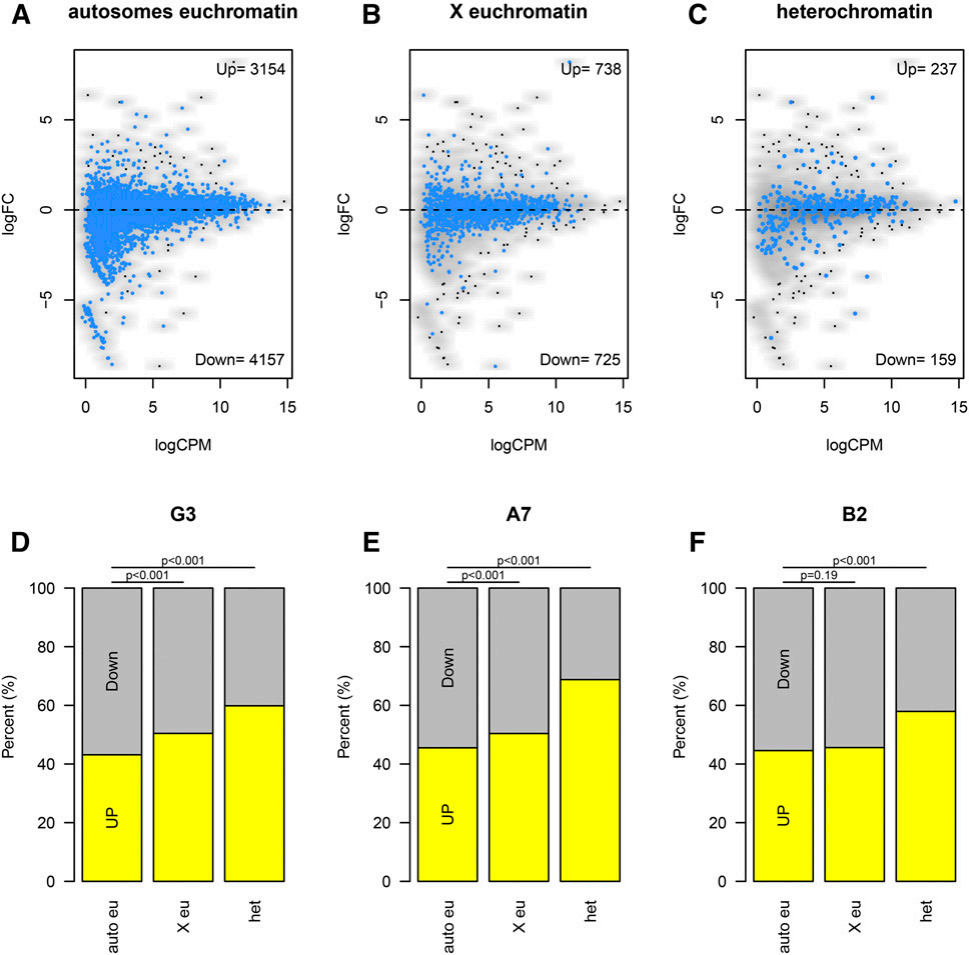
Increased transcription of heterochromatin and X-linked genes in *upSET* mutant cell lines. (A) Scatter plot showing fold-change for individual genes based on their nascent-RNA levels in the G3 *upSET* mutant cell line on the *y*-axis *vs.* wild type S2 expression level on the *x*-axis. Autosomal euchromatin genes are highlighted in blue against all other genes. Euchromatin or heterochromatin annotations were based on H3K9me2 levels in S2 cells. (B) As in (A), except with X chromosome euchromatin genes highlighted. (C) As in (A and B), except with heterochromatin genes highlighted. (D) Summary of (A–C) with number of genes falling above and below the zero-fold change line from the G3 *upSET* mutant cell line. The general trends of increased expression on the X chromosome and of heterochromatin genes are statistically significant (*P* < 0.001). (E) As in (D), except using data comparing the A7 *upSET* mutant cell line to S2 cells. The same general trends are observed as in (D). (F) As in (D and E), except using data comparing the B2 *upSET* mutant cell line to S2 cells. The same general trends are observed as in (D and E); however, upregulation of the X chromosome was not observed in the B2 line (*P* = 0.19).

Statistical analysis to identify upregulated or downregulated genes revealed striking heterogeneity in the nascent-RNA-sequencing transcriptional profiles between the three *upSET*-mutant cell lines. Therefore, we broadened our analysis to identify differential trends for groups of genes rather than individual loci, based on their previously determined chromatin environment or genomic location, for example, in heterochromatin *vs.* euchromatin, or X-linked *vs.* autosomal. To do so, we counted the numbers of genes falling above and below the no-fold-change (log_2_FC = 0) line per grouping (Figure 6, A–C). When comparing X-linked *vs.* autosomal genes, there was a weak trend toward upregulation, though this failed to reach statistical significance in all three *upSET* mutant cell lines (Figure 6, D–F, second bar). The most striking trend, which was consistent for all three *upSET* mutant cell lines, was upregulation of heterochromatin genes, as defined by the presence of H3K9me2 over the gene in wild-type S2 cells (Figure 6, D–F, third bar). While the fold change for individual genes generally was not statistically significant, taken as a whole, the distribution of the heterochromatin genes skewed toward increased expression in a statistically significant manner.

In summary, we found that the loss of UpSET has a consistent effect on heterochromatin composition and function, based on multiple assays in flies and in cell culture. Since it has been proposed that MSL proteins play a role in activation of autosomal heterochromatin genes in males (Koya and Meller 2015), it is possible that the heterochromatin phenotype that we observe is due, at least in part, to disruption of a specific interaction between UpSET and the MSL complex. However, given the previous study showing derepression of silent genes and repetitive transposable elements in ovaries and female Kc cells (Rincon-Arano *et al.* 2012), along with our finding that UpSET is an essential gene, it is likely that UpSET plays a broad role in maintaining the balance between active and silent marks in both sexes.

## Discussion

Chromatin and gene expression are intimately linked at the molecular level. Proteins that create and maintain chromatin domains therefore are critical for transcriptional fidelity of gene expression programs. Here, we have further investigated one such chromatin protein, the SET-domain containing protein UpSET. Previous characterizations of this protein and its homologs SET3 in yeast, and MLL5 in mammals, have shown that it assembles into a complex with histone deacetylase activity (Pijnappel *et al.* 2001). Furthermore, the PHD finger of UpSET has been shown to interact with the histone post-translational modification H3K4me2/3 (Ali *et al.* 2013; Lemak *et al.* 2013), which results in recruitment of the HDAC complex to TSS-proximal locations. Once there, the HDAC complex restricts the spread of activating marks which prevents the improper activation of neighboring genes (Kim and Buratowski 2009; Kim *et al.* 2012; Rincon-Arano *et al.* 2012). Our results are largely compatible with this model, yet suggest that this role may be particularly important for the maintenance of the heterochromatin environment.

The original characterization of UpSET in *Drosophila* made use of a *P*-element insertion line, which left the coding region of the gene intact, and was described as homozygous viable with female sterility. Additionally, many of the experiments were performed in female cultured cells and in female tissues (Rincon-Arano *et al.* 2012). Interestingly, our laboratory independently discovered UpSET as one of the most enriched proteins in crosslinked MSL3 purifications. This led us to seek whether UpSET plays a role in dosage compensation, a male-specific process in *Drosophila*.

To our surprise, we found that precise deletion of the *upSET* locus was lethal in both males and females. That we were able to rescue this lethality with a tagged UpSET transgene suggests that it is due specifically to the loss of UpSET. Furthermore, it suggests that the previously utilized *P*-element allele may be hypomorphic, and still provide enough UpSET protein for viability, but then result in maternal effect lethality as characterized. Using our tagged allele, we determined the genomic localization of UpSET protein in mixed embryos, confirming, while also refining, the previous result for UpSET localization to active genes by Dam-ID (Rincon-Arano *et al.* 2012).

In parallel, we created S2 male cell lines that carried *upSET* mutations that introduce frameshifts to the UpSET open reading frame. The cell culture system proved invaluable for assessing the molecular impact of the loss of UpSET. We observed that loss of UpSET had a profound impact on the state of chromatin, which in broad strokes was consistent across all three cell lines. Consistent with a role related to deacetylation, we saw a modest increase in acetylated histone H4 in bulk histones, but the more striking change was that H3K9me2 levels in bulk histones were reduced in all three lines. In addition, our nascent-RNA-sequencing showed increases in transcription of genes embedded in heterochromatin regions in all three lines. These molecular findings were further supported by our analysis of *upSET*-deficient flies when we tested the impact of the heterozygous *upSET* deletion on the position effect variegation phenotype of the *w^m4^* allele. Our findings showed suppressed *white* variegation, suggesting a loss of heterochromatin stability allowing the *white* locus to become expressed more readily.

Interestingly, there has long been an, as yet unexplained, relationship between heterochromatin and the X chromosome in *Drosophila*. The X chromosome is observed to be less compact in polytene chromosome preparations, and its morphology is particularly sensitive to mutations in heterochromatin components such as Su(var)3–7, Su(var)3–9, and HP1a (Demakova *et al.* 2007; Spierer *et al.* 2008). Loss of these core heterochromatin factors leads to a swollen X chromosome (Demakova *et al.* 2007), whereas their overexpression can lead to enhanced compaction, as compared to changes in autosomes (Spierer *et al.* 2008). Furthermore, Jil-1 kinase, which is enriched ∼2-fold on the male X in an MSL-dependent manner, is thought to prevent the spread of heterochromatin by catalyzing the H3S10ph modification (Jin *et al.* 1999, 2000). Jil-1 has a complex interplay with heterochromatin components (Ebert *et al.* 2004; Deng *et al.* 2005, 2007), with evidence for roles in phosphorylation of Su(var)3–9 (Boeke *et al.* 2010), and for establishing a composite H3S10phK9me2 epigenetic mark (Wang *et al.* 2014). Indeed, using *Jil-1* mutant larvae, it has been observed that H3K9me2 spreads from pericentric heterochromatin into the euchromatic gene arms, with a marked increase on the X chromosome in both sexes (A. Plachetka, A. Alekseyenko, and M.I.K., modENCODE, unpublished observations). Intriguingly this spread appeared to skip over gene bodies, suggesting additional non-Jil-1 mechanisms exist for protecting genes from the spread of heterochromatin.

The unique function of UpSET in heterochromatin in *Drosophila* may make sense in terms of evolutionary history, since the mammalian homolog MLL5 has been implicated in establishing proper DNA methylation. Canonical (5-methylcytosine) DNA methylation is a repressed state found in higher eukaryotes, but is found at only very low levels in *Drosophila* (<1%) and is of uncertain significance in flies (Capuano *et al.* 2014; Takayama *et al.* 2014). However, the exact molecular role for MLL5 with respect to DNA methylation has not been elucidated (Yun *et al.* 2014). One can posit that this derived trait in mammals is an extension of the repressive role of SET3 in yeast, with UpSET functioning in an intermediate manner. This expanding alternative repressive role may explain how this family of atypical catalytically inactive SET-domain proteins has perdured through evolutionary time.

## Supplementary Material

Supplemental material is available online at www.g3journal.org/lookup/suppl/doi:10.1534/g3.116.037788/-/DC1.
