# Supplementary material for: upSET, the Drosophila homologue of SET3, Is Required for Viability and the Proper Balance of Active and Repressive Chromatin Marks
Source: G3 (Bethesda). 2017 Jan 4;7(2):625–35. doi: 10.1534/g3.116.037788 (PMC5295607; doi:10.1534/g3.116.037788)
Supplement: Supplementary file 1 [file 625FigureS1.pptx]

## Slide 1
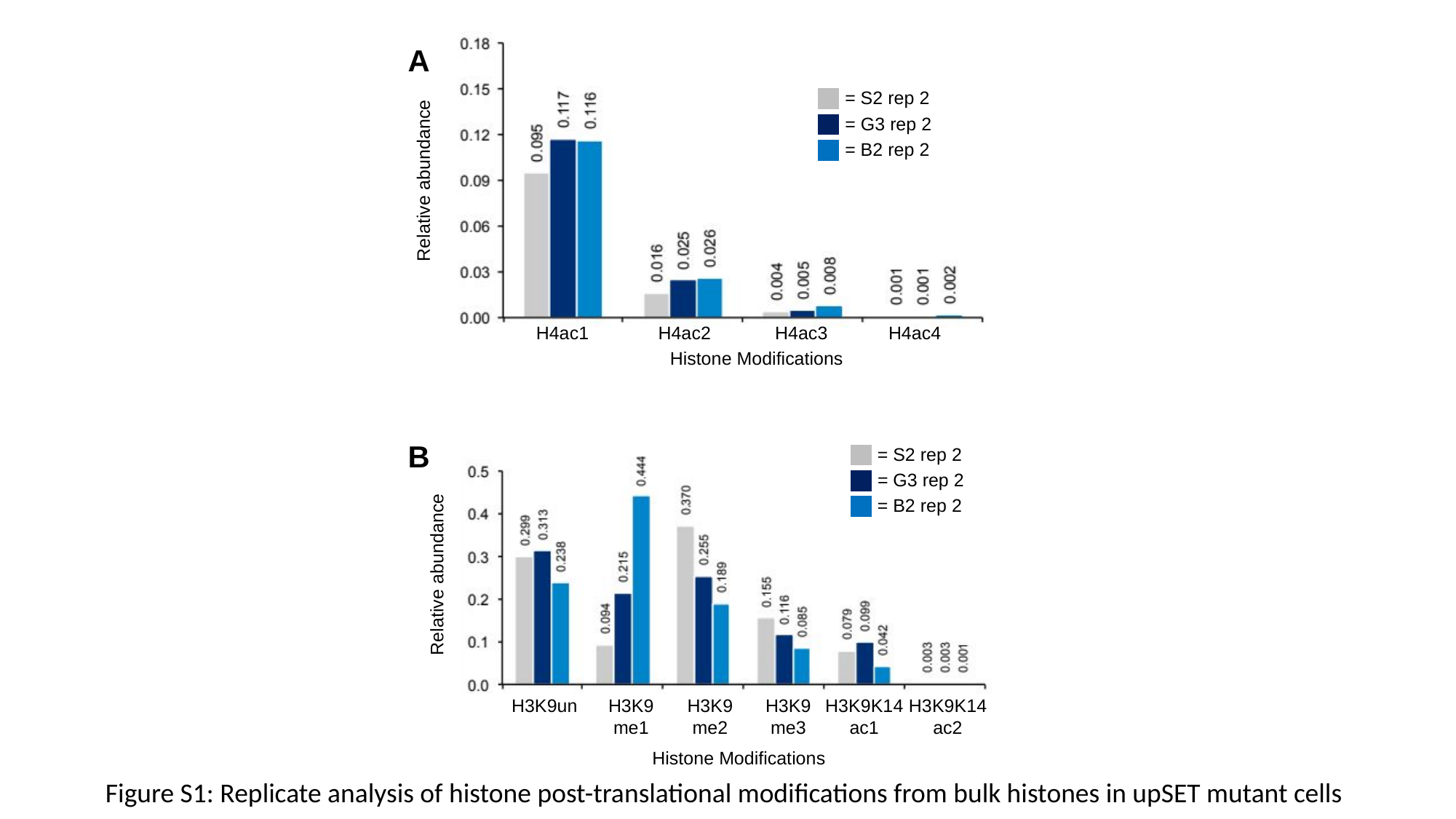

A
= S2 rep 2
= G3 rep 2
= B2 rep 2
Relative abundance
H4ac1
H4ac2
H4ac3
H4ac4
Histone Modifications
B
= S2 rep 2
= G3 rep 2
= B2 rep 2
Relative abundance
H3K9un
H3K9
me1
H3K9
me2
H3K9
me3
H3K9K14
ac1
H3K9K14
ac2
Histone Modifications
Figure S1: Replicate analysis of histone post-translational modifications from bulk histones in upSET mutant cells
