# Supplementary material for: upSET, the Drosophila homologue of SET3, Is Required for Viability and the Proper Balance of Active and Repressive Chromatin Marks
Source: G3 (Bethesda). 2017 Jan 4;7(2):625–35. doi: 10.1534/g3.116.037788 (PMC5295607; doi:10.1534/g3.116.037788)
Supplement: Supplementary file 2 [file 625FigureS2.pptx]

## Slide 1
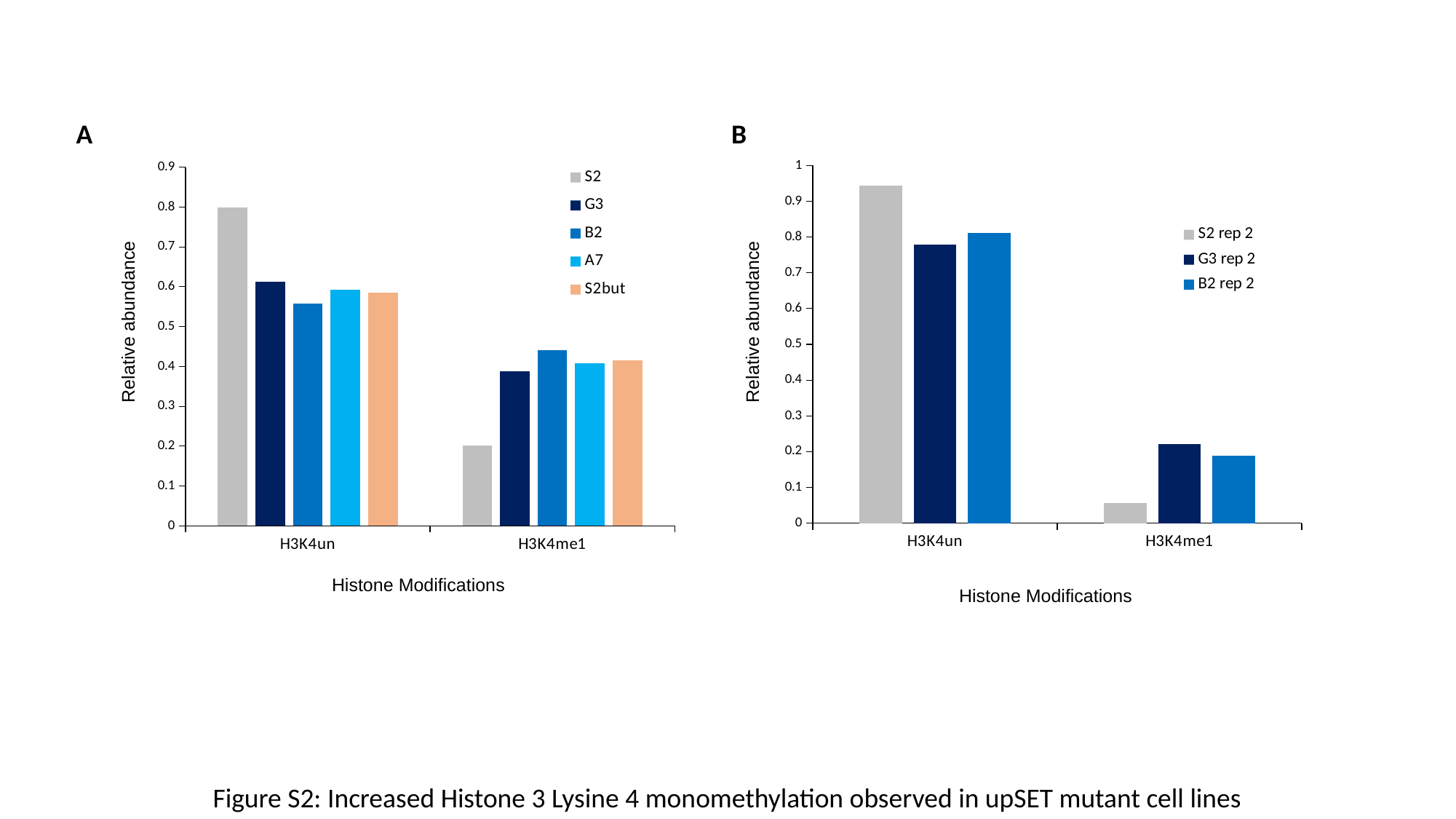

A
B
### Chart
| Category | S2 | G3 | B2 | A7 | S2but |
|---|---|---|---|---|---|
| H3K4un | 0.7989137170482523 | 0.6119982122025834 | 0.5583209535995751 | 0.5927601566143489 | 0.585329073557641 |
| H3K4me1 | 0.20108628295174769 | 0.3880017877974166 | 0.4416790464004249 | 0.4072398433856511 | 0.41467092644235903 |
### Chart
| Category | | | |
|---|---|---|---|
| H3K4un | 0.9433510098927474 | 0.779249885862278 | 0.8113273014007755 |
| H3K4me1 | 0.05664899010725255 | 0.22075011413772203 | 0.1886726985992245 |Relative abundance
Relative abundance
Histone Modifications
Histone Modifications
Figure S2: Increased Histone 3 Lysine 4 monomethylation observed in upSET mutant cell lines
