# Supplementary material for: upSET, the Drosophila homologue of SET3, Is Required for Viability and the Proper Balance of Active and Repressive Chromatin Marks
Source: G3 (Bethesda). 2017 Jan 4;7(2):625–35. doi: 10.1534/g3.116.037788 (PMC5295607; doi:10.1534/g3.116.037788)
Supplement: Supplementary file 3 [file 625FigureS3.pptx]

## Slide 1
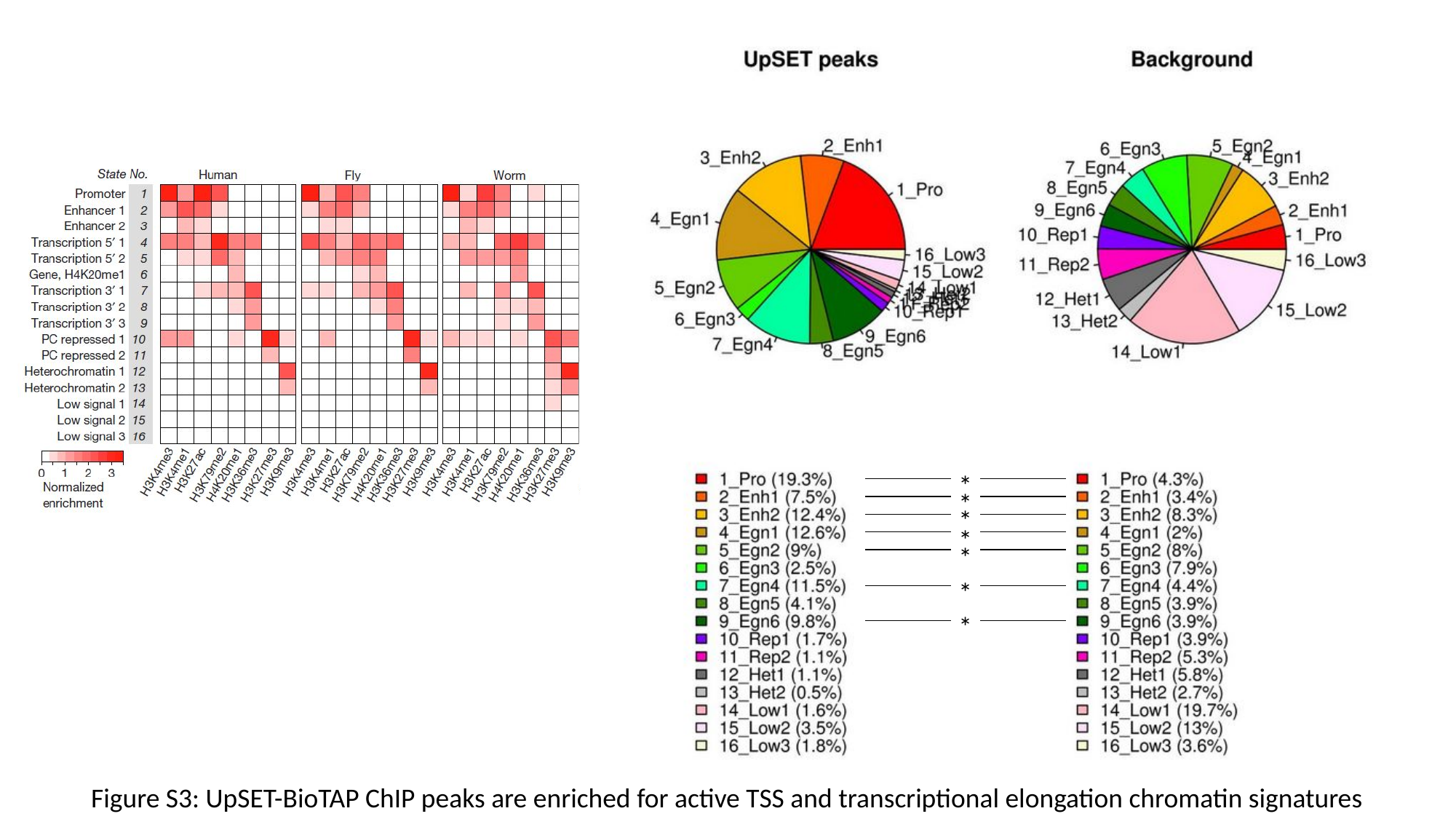

*
*
*
*
*
*
*
Figure S3: UpSET-BioTAP ChIP peaks are enriched for active TSS and transcriptional elongation chromatin signatures
