# Supplementary material for: upSET, the Drosophila homologue of SET3, Is Required for Viability and the Proper Balance of Active and Repressive Chromatin Marks
Source: G3 (Bethesda). 2017 Jan 4;7(2):625–35. doi: 10.1534/g3.116.037788 (PMC5295607; doi:10.1534/g3.116.037788)
Supplement: Supplementary file 4 [file 625FigureS4.pptx]

## Slide 1
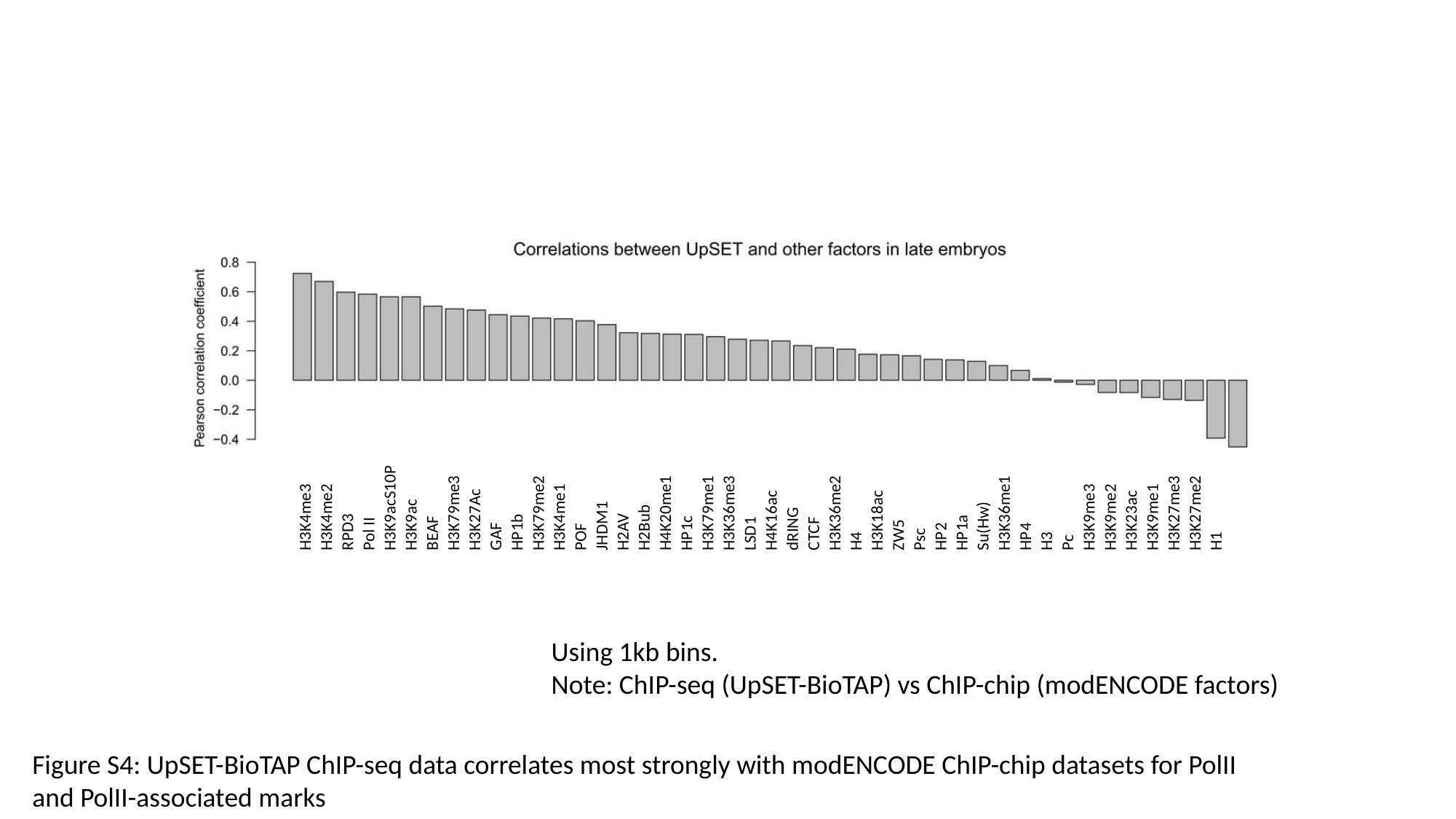

H3K4me3
H3K4me2
RPD3
Pol II
H3K9acS10P
H3K9ac
BEAF
H3K79me3
H3K27Ac
GAF
HP1b
H3K79me2
H3K4me1
POF
JHDM1
H2AV
H2Bub
H4K20me1
HP1c
H3K79me1
H3K36me3
LSD1
H4K16ac
dRING
CTCF
H3K36me2
H4
H3K18ac
ZW5
Psc
HP2
HP1a
Su(Hw)
H3K36me1
HP4
H3
Pc
H3K9me3
H3K9me2
H3K23ac
H3K9me1
H3K27me3
H3K27me2
H1
Using 1kb bins.
Note: ChIP-seq (UpSET-BioTAP) vs ChIP-chip (modENCODE factors)
Figure S4: UpSET-BioTAP ChIP-seq data correlates most strongly with modENCODE ChIP-chip datasets for PolII and PolII-associated marks
