# Supplementary figures and images for: upSET, the Drosophila homologue of SET3, Is Required for Viability and the Proper Balance of Active and Repressive Chromatin Marks
Source: G3 (Bethesda). 2017 Jan 4;7(2):625–35. doi: 10.1534/g3.116.037788 (PMC5295607; doi:10.1534/g3.116.037788)

## Slide 1
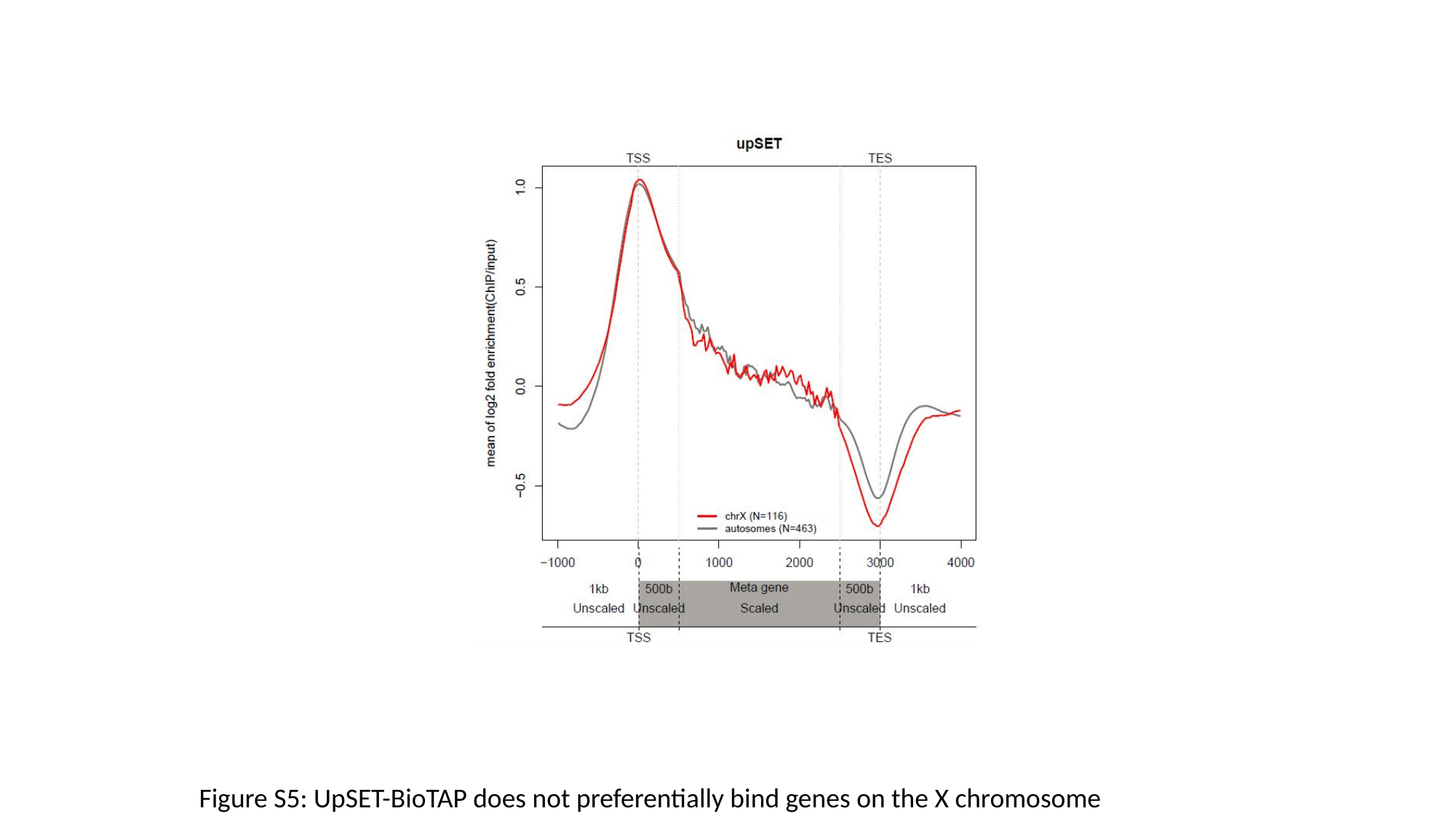

Figure S5: UpSET-BioTAP does not preferentially bind genes on the X chromosome

Supplement: Supplementary file 5 [file 625FigureS5.pptx]
