# Supplementary material for: upSET, the Drosophila homologue of SET3, Is Required for Viability and the Proper Balance of Active and Repressive Chromatin Marks
Source: G3 (Bethesda). 2017 Jan 4;7(2):625–35. doi: 10.1534/g3.116.037788 (PMC5295607; doi:10.1534/g3.116.037788)
Supplement: Supplementary file 6 [file 625FigureS6.pptx]

## Slide 1
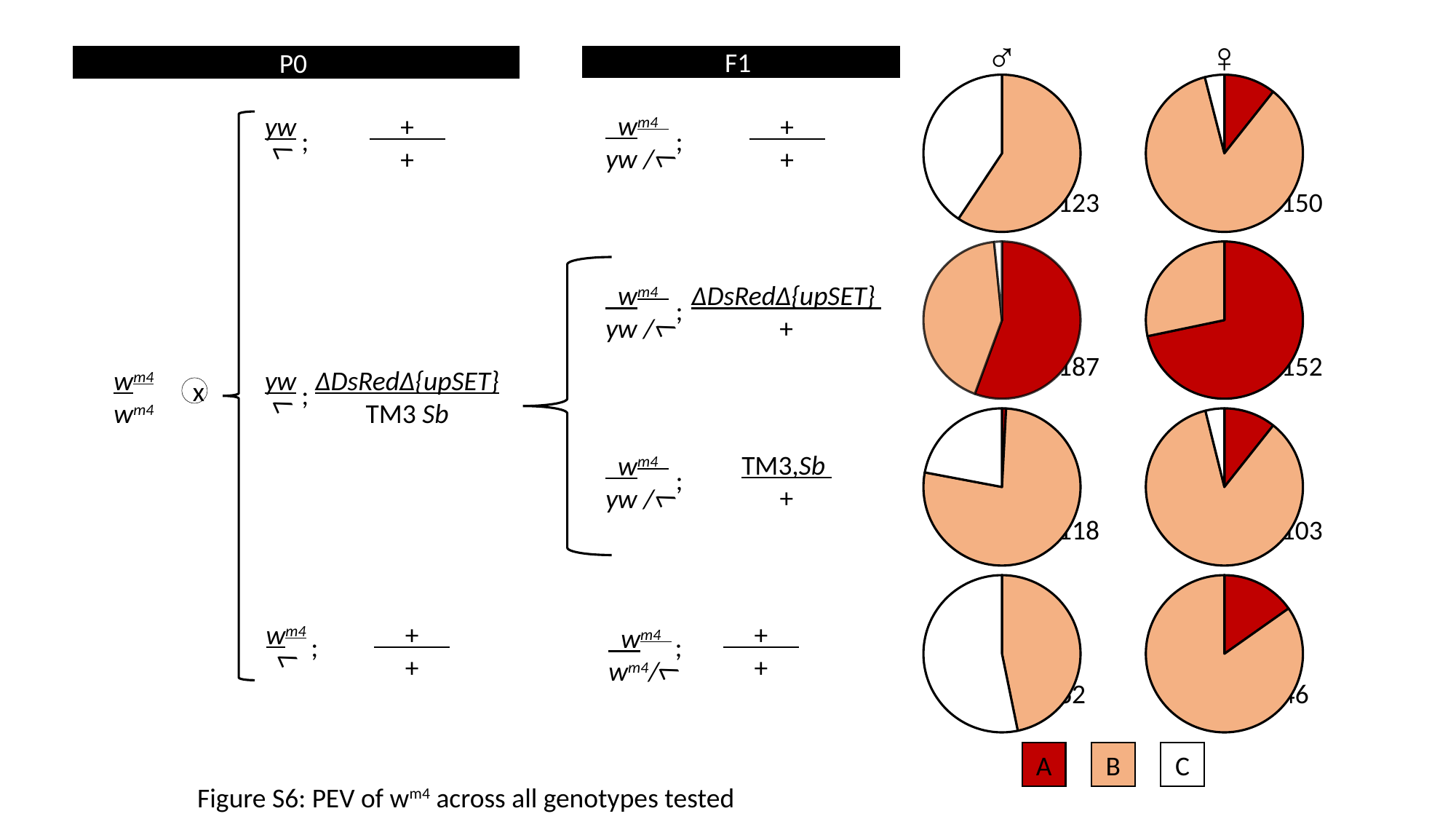

♂
♀
### Chart
| Category | |
|---|---|
### Chart
| Category | |
|---|---|
### Chart
| Category | |
|---|---|
### Chart
| Category | |
|---|---|
### Chart
| Category | |
|---|---|
### Chart
| Category | |
|---|---|
### Chart
| Category | |
|---|---|
### Chart
| Category | |
|---|---|P0
F1
 wm4
yw /
7
 +
+
;
yw
7
 +
+
;
123
187
118
62
150
152
103
46
 wm4
yw /
7
ΔDsRedΔ{upSET}
+
;
TM3,Sb
+
 wm4
yw /
7
;
wm4
wm4
yw
7
ΔDsRedΔ{upSET}
TM3 Sb
;
x
 +
+
wm4
7
;
 +
+
 wm4
wm4/
7
;
A
B
C
Figure S6: PEV of wm4 across all genotypes tested
