# Supplementary material for: upSET, the Drosophila homologue of SET3, Is Required for Viability and the Proper Balance of Active and Repressive Chromatin Marks
Source: G3 (Bethesda). 2017 Jan 4;7(2):625–35. doi: 10.1534/g3.116.037788 (PMC5295607; doi:10.1534/g3.116.037788)
Supplement: Supplementary file 7 [file 625TableS1.docx]

**Table S1. *upSET* gRNA oligos and HRMA Primers**

| **Name** | **Sequence** | **Description** |
| --- | --- | --- |
| **Crispr_upSET_1F** | gttcgAACCGAGTCGTGACTGGACA | 3L:14000954..14000976 (-strand) CRISPR seq: AACCGAGTCGTGACTGGACATGG; remove NGG add BbsI cloning site fragments to generate oligos at left;  no off targets, efficiency score = 3.96; 4bp downstream from ATG |
| **Crispr_upSET_1R** | aaacTGTCCAGTCACGACTCGGTTc |  |
| **Crispr_upSET_3F** | gttcgAGGCGCGATGCCGTCTGATT | 3L:14010960..14010982 (+ strand) CRISPR seq: AGGCGCGATGCCGTCTGATTAGG; remove NGG add BbsI cloning site fragments to generate oligos at left;  no off targets, efficiency score = 7.71; at stop codon |
| **Crispr_upSET_3R** | aaacAATCAGACGGCATCGCGCCTc |  |
| **Crispr_upSET_5F** | gttcgTGGCCAGGCGCAGTAGTAAT | 3L:13995983..13996005 (+ strand) CRISPR seq: TGGCCAGGCGCAGTAGTAATAGG; remove NGG add BbsI cloning site fragments to generate oligos at left;  no off targets, efficiency score = 7.08; 5'UTR, ~5kb upstream of ATG |
| **Crispr_upSET_5R** | aaacATTACTACTGCGCCTGGCCAc |  |
| **Crispr_upSET_7F** | gttcgACAGCAGATCAGCCTACCGC | 3L:14002035..14002057 (+ strand) CRISPR seq: ACAGCAGATCAGCCTACCGCAGG; remove NGG add BbsI cloning site fragments to generate oligos at left;  no off targets, efficiency score = 6.55; exon 1, ~1kb downstream from start |
| **Crispr_upSET_7R** | aaacGCGGTAGGCTGATCTGCTGTc |  |
| **KAM187** | ccactgggagtttcagcttc | ~250bp left of Crispr_upSET_1 target; HRMA primer set step 1 |
| **KAM188** | gcgactgattgatcgactga | ~250bp right of Crispr_upSET_1; HRMA primer set step 1 |
| **KAM201** | gctgcacatgtttgatgataagc | ~250bp left of Crispr_upSET_3; HRMA primer set step 1 |
| **KAM202** | gtgcaagctcatactttatgcgc | ~250bp right of Crispr_upSET_3; HRMA primer set step 1 |
| **KAM203** | gcactcttcggcagtatggt | ~250bp left of Crispr_upSET_5; HRMA primer set step 1 |
| **KAM204** | cgtatggcacaagaagcaga | ~250bp right of Crispr_upSET_5; HRMA primer set step 1 |
| **KAM205** | ccttccctgtaaacacacgtc | ~50bp left of Crispr_upSET_1 target; HRMA primer set step 2 |
| **KAM206** | cgatgcgatttatgctgctgg | ~50bp right of Crispr_upSET_1; HRMA primer set step 2 |
| **KAM209** | ggtcatcgagcgattggg | ~50bp left of Crispr_upSET_3; HRMA primer set step 2 |
| **KAM210** | ggattgaaaggcattcaattaagac | ~50bp right of Crispr_upSET_3; HRMA primer set step 2 |
| **KAM211** | cgaataggcggaaaggcg | ~50bp left of Crispr_upSET_5; HRMA primer set step 2 |
| **KAM212** | gccctgcttcttcttcttgg | ~50bp right of Crispr_upSET_5; HRMA primer set step 2 |
| **KAM236** | cagcagcagcaactactacag | ~250bp left of Crispr_upSET_7 target; HRMA primer set step 1 |
| **KAM237** | ggttatcagtgaggagttcgc | ~250bp right of Crispr_upSET_7; HRMA primer set step 1 |
| **KAM250** | caatggtcaccacgtcgac | ~50bp left of Crispr_upSET_7 target; HRMA primer set step 2 |
| **KAM251** | gagactgctgcagaagatatcc | ~50bp right of Crispr_upSET_7; HRMA primer set step 2 |

Oligonucleotide pairs for cloning of *upSET*-targeted gRNA constructs into pL018 and a description of the CRISPR sequence location are listed. Primers used for HRMA assays to identify potential mutant cell lines and subsequent sequence verification of mutations are also listed. All sequences are written from 5’ to 3’.
