# Supplementary material for: upSET, the Drosophila homologue of SET3, Is Required for Viability and the Proper Balance of Active and Repressive Chromatin Marks
Source: G3 (Bethesda). 2017 Jan 4;7(2):625–35. doi: 10.1534/g3.116.037788 (PMC5295607; doi:10.1534/g3.116.037788)
Supplement: Supplementary file 8 [file 625FileS1.docx]

**Supplemental Figure Legends**

Figure S1: Replicate analysis of histone post-translational modifications from bulk histones in upSET mutant cells

1. Relative quantification (1.0 = 100%) of H4K5K8K12K16 mono-, di-, tri-, and tetra-acetyl patterns in *upSET* mutant cell lines (G3 and B2) and the parental S2 line. Trends observed with these replicates are the same as in Figure 2.
2. Relative quantification (1.0 = 100%) of H3K9K14 PTM patterns in *upSET* mutant cell lines (G3 and B2) and the parental S2 line. The H3K9me2/me3 marks are depleted from *upSET* mutant S2 cells, suggestive of an effect on heterochromatin. Trends observed with these replicates are the same as in Figure 2.

Figure S2: Increased Histone 3 Lysine 4 monomethylation observed in upSET mutant cell lines

1. Relative quantification (1.0 = 100%) of H3K4 monomethylation in *upSET* mutant cell lines (G3, B2, and A7), butyrate treated cells (S2but), and the parental S2 line. The biochemical properties of H3K4me2/me3 make these marks impossible to quantify under these experimental conditions.
2. Replicate analysis of H3K4 monomethylation in *upSET* mutant cell lines (G3 and B2) and the parental S2 line. The same general trend of increased H3K4me1 in *upSET* mutant cells is observed as in (A).

Figure S3: UpSET-BioTAP ChIP peaks are enriched for active TSS and transcriptional elongation chromatin signatures

Ho et al (2014) used eight histone post-translational modifications across three species and machine learning to identify 16 different classes of chromatin signatures (left panel). UpSET-BioTAP peaks largely fall into the active signatures, with the highest enrichment of promoter regions (state 1). UpSET-BioTAP peaks also enrich for enhancer and transcription signatures (states 2, 3, 4, 5, 7, and 9) as compared to the genome-wide background (*p<0.001, permutation test). Shorthand used in the right panel are as follows: Pro = Promoter, Enh = Enhancer, Egn = Expressed Gene, Rep = Polycomb Group Repressed, Het = Heterochromatin, Low = Low signal. Figure S3 is related to Figure 4.

Figure S4: UpSET-BioTAP ChIP-seq data correlate most strongly with modENCODE ChIP-chip datasets for PolII and PolII-associated marks

Pearson correlation coefficients were calculated for the pairwise comparison of the UpSET-BioTAP ChIP-seq with a number of ChIP-chip profiles for chromosomal proteins generated by the modENCODE project using 1kb windows. The highest degrees of correlation were found with proteins and histone PTMs associated with active genes, such as RNA polymerase II, H3K4me3, and H3K4me2. It should be noted that the difference in platform (UpSET-BioTAP Chip-seq vs ChIP-chip for modENCODE factors) may artificially depress the Pearson correlation coefficient values. Figure S4 is related to Figure 4.

Figure S5: UpSET-BioTAP does not preferentially bind genes on the X chromosome

Metagene profile from mixed male and female UpSET-BioTAP-expressing transgenic embryos comparing the pattern of UpSET-BioTAP across the gene bodies (scaling as in Figure 4B) of genes on the X versus the Autosomes. No major differential pattern is detected from the UpSET-BioTAP ChIP-seq from mixed embryos.

Figure S6: Position effect variegation of *w^m4^* for all genotypes tested

Females of the genotype *w^m4^*/*w^m4^* were crossed to males of three different genotypes as indicated in the P_0_ generation. The *yw* cross is the control cross; the *w^m4^* data is from the stock and is listed for reference. F_1_ progeny were sorted based on sex and presence of balancer chromosome markers. After three days, flies were scored for their eye phenotype into 3 class (see Figure 5B). The indicated total number of progeny scored per genotype accompany the pie chart. Pairwise comparisons using the Chi-squared test indicate that the control cross is not statistically different than the distribution of PEV naturally occurring in the *w^m4^* stock (p=0.10, p=0.29, for males and females, respectively). Heterozygous loss of *upSET* does result in suppressed variegation as compared to the control cross (Chi-squared, p=7e-31, p=3e-26, for males and females, respectively). Figure S6 is related to Figure 5.

**Supplemental References**

Ho, J. W., Y. L. Jung, T. Liu, B. H. Alver, S. Lee *et al.*, 2014 Comparative analysis of metazoan chromatin organization. Nature 512: 449-452.
